# Predicting Individual Pain Sensitivity Using a Novel Cortical Biomarker Signature

**DOI:** 10.1001/jamaneurol.2024.4857

**Published:** 2025-01-27

**Authors:** Nahian S. Chowdhury, Chuan Bi, Andrew J. Furman, Alan K. I. Chiang, Patrick Skippen, Emily Si, Samantha K. Millard, Sarah M. Margerison, Darrah Spies, Michael L. Keaser, Joyce T. Da Silva, Shuo Chen, Siobhan M. Schabrun, David A. Seminowicz

**Affiliations:** 1 Center for Pain IMPACT, Neuroscience Research Australia, Sydney, New South Wales, Australia; 2 University of New South Wales, Sydney, New South Wales, Australia; 3 Division of Biostatistics and Bioinformatics, Department of Epidemiology and Public Health, University of Maryland School of Medicine, Baltimore; 4 Division of Biostatistics, Center for Devices and Radiological Health, US Food and Drug Administration, Silver Spring, Maryland; 5 Department of Neural and Pain Sciences, University of Maryland School of Dentistry, Baltimore; 6 Center to Advance Chronic Pain Research, University of Maryland, Baltimore; 7 Data Sciences, Hunter Medical Research Institute, Newcastle, New South Wales, Australia; 8 School of Medicine and Public Health, University of Newcastle, Newcastle, New South Wales, Australia; 9 The Gray Centre for Mobility and Activity, Parkwood Institute, St Joseph’s Healthcare, London, Ontario, Canada; 10 School of Physical Therapy, University of Western Ontario, London, Ontario, Canada; 11 Department of Medical Biophysics, Schulich School of Medicine & Dentistry, University of Western Ontario, London, Ontario, Canada

## Abstract

**Importance:**

Biomarkers would greatly assist decision-making in the diagnosis, prevention, and treatment of chronic pain.

**Objective:**

To undertake analytical validation of a sensorimotor cortical biomarker signature for pain consisting of 2 measures: sensorimotor peak alpha frequency (PAF) and corticomotor excitability (CME).

**Design, Setting, and Participants:**

This cohort study at a single center (Neuroscience Research Australia) recruited participants from November 2020 to October 2022 through notices placed online and at universities across Australia. Participants were healthy adults aged 18 to 44 years with no history of chronic pain or a neurological or psychiatric condition. Participants experienced a model of prolonged temporomandibular pain with outcomes collected over 30 days. Electroencephalography to assess PAF and transcranial magnetic stimulation (TMS) to assess CME were recorded on days 0, 2, and 5. Pain was assessed twice daily from days 1 through 30.

**Exposure:**

Participants received an injection of nerve growth factor (NGF) to the right masseter muscle on days 0 and 2 to induce prolonged temporomandibular pain lasting up to 4 weeks.

**Main Outcomes and Measures:**

The predictive accuracy of the PAF/CME biomarker signature was determined using a nested control-test scheme: machine learning models were run on a training set (n = 100), where PAF and CME were predictors and pain sensitivity was the outcome. The winning classifier was assessed on a test set (n = 50) comparing the predicted pain labels against the true labels.

**Results:**

Among the final sample of 150 participants, 66 were female and 84 were male; the mean (SD) age was 25.1 (6.2) years. The winning classifier was logistic regression, with an outstanding area under the curve (AUC = 1.00). The locked model assessed on the test set had excellent performance (AUC = 0.88; 95% CI, 0.78-0.99). Results were reproduced across a range of methodological parameters. Moreover, inclusion of sex and pain catastrophizing as covariates did not improve model performance, suggesting the model including biomarkers only was more robust. PAF and CME biomarkers showed good to excellent test-retest reliability.

**Conclusions and Relevance:**

This study provides evidence for a sensorimotor cortical biomarker signature for pain sensitivity. The combination of accuracy, reproducibility, and reliability suggests the PAF/CME biomarker signature has substantial potential for clinical translation, including predicting the transition from acute to chronic pain.

## Key Points

**Question:**

Can individuals be accurately classified as having high or low pain sensitivity based on 2 features of cortical activity, sensorimotor peak alpha frequency (PAF) and corticomotor excitability (CME)?

**Findings:**

In a cohort study involving 150 healthy participants, the performance of a logistic regression model was outstanding in a training set (n = 100) and excellent in a test set (n = 50), with the combination of slower PAF and CME depression predicting higher pain. Results were reproduced across a range of methodological parameters.

**Meaning:**

A novel cortical biomarker can accurately distinguish high and low pain-sensitive individuals and may predict the transition from acute to chronic pain.

## Teaser

This cohort study analyzes electroencephalography data from healthy participants to analyze and validate a cortical biomarker signature for pain, which comprised the measures sensorimotor peak alpha frequency and corticomotor excitability.

## Introduction

Several objective pain biomarkers have been proposed, including neuroimaging markers of mechanistic/structural abnormalities,^1,2,3^ neural oscillatory rhythms^4^ and “multi-omics” metrics of micro RNA,^5^ proteins,^6^ lipids, and metabolites.^7^ Such biomarkers would greatly assist decision-making in the diagnosis, prevention, and treatment of chronic pain.^8^ However, attempts at establishing pain biomarkers have suffered from either insufficient sample sizes to conduct full-scale analytical validation using machine learning,^8,9,10^ failure to use clinically relevant pain models,^11,12,13^ or lack of assessment of reproducibility or test-retest reliability.^14,15^ These factors have hindered the clinical translatability of prospective pain biomarkers.

Research suggests that the neural oscillatory rhythms involved in processing nociceptive input, and the corticospinal signaling involved in the subsequent motor response, are both critical in shaping the subjective experience of pain.^4,16^ This work has culminated to the identification of a promising sensorimotor cortical biomarker signature for predicting pain sensitivity involving 2 metrics: (1) sensorimotor peak alpha frequency (PAF), defined as the dominant sensorimotor cortical oscillation in the alpha (8-12 Hz) range,^17^ and (2) corticomotor excitability (CME), defined as the efficacy at which signals are relayed from primary motor cortex (M1) to peripheral muscles.^18^ Previous work has shown that slower PAF before pain onset and reduced CME during prolonged pain (“depression”) are associated with more pain, while faster PAF and increased CME (“facilitation”) are associated with less pain.^19,20,21,22,23^ Given that individuals who experience higher pain in the early stages of a prolonged pain episode (eg, postsurgery) are more likely to develop chronic pain in the future,^24^ slow PAF before an anticipated prolonged pain episode and/or CME depression during the acute stages of pain could be predictors for the transition to chronic pain.

This article presents the main outcomes of the PREDICT trial, a preregistered (NCT04241562^25^) analytical validation of the PAF/CME biomarker signature using a model of prolonged myofascial temporomandibular pain (masseter intramuscular injection of nerve growth factor [NGF]). Repeated NGF injections induce progressively developing prolonged pain lasting up to 4 weeks^23,26^ and has been shown to mimic chronic pain characteristics such as time course (gradual development), type of pain (movement evoked), functional impairments, hyperalgesia (decreased pressure pain thresholds), and mechanism of sensitization.^27,28^ This makes the NGF model a highly standardized prolonged pain model with which to undertake biomarker validation.

The aim of the PREDICT trial was to determine whether individuals could be accurately classified as having high or low pain sensitivity based on baseline PAF and CME facilitator/depressor classification. We predicted the area under the curve (AUC) of the receiver operator characteristic (ROC) curve for distinguishing high and low pain-sensitive individuals would be at least 70% (which represents an acceptable AUC).^29^

## Methods

### Participants

Ethical approval for the PREDICT trial was obtained from the University of New South Wales (HC190206) and the University of Maryland Baltimore (HP-00085371). Written informed consent was obtained. The eMethods in Supplement 1 contains all additional details regarding participant characteristics and methodology (see also eTables 1 and 2 and eFigures 1 and 2 in Supplement 1). We followed the Strengthening the Reporting of Observational Studies in Epidemiology (STROBE) reporting guidelines for cohort studies.

### Experimental Protocol

**Figure 1.**
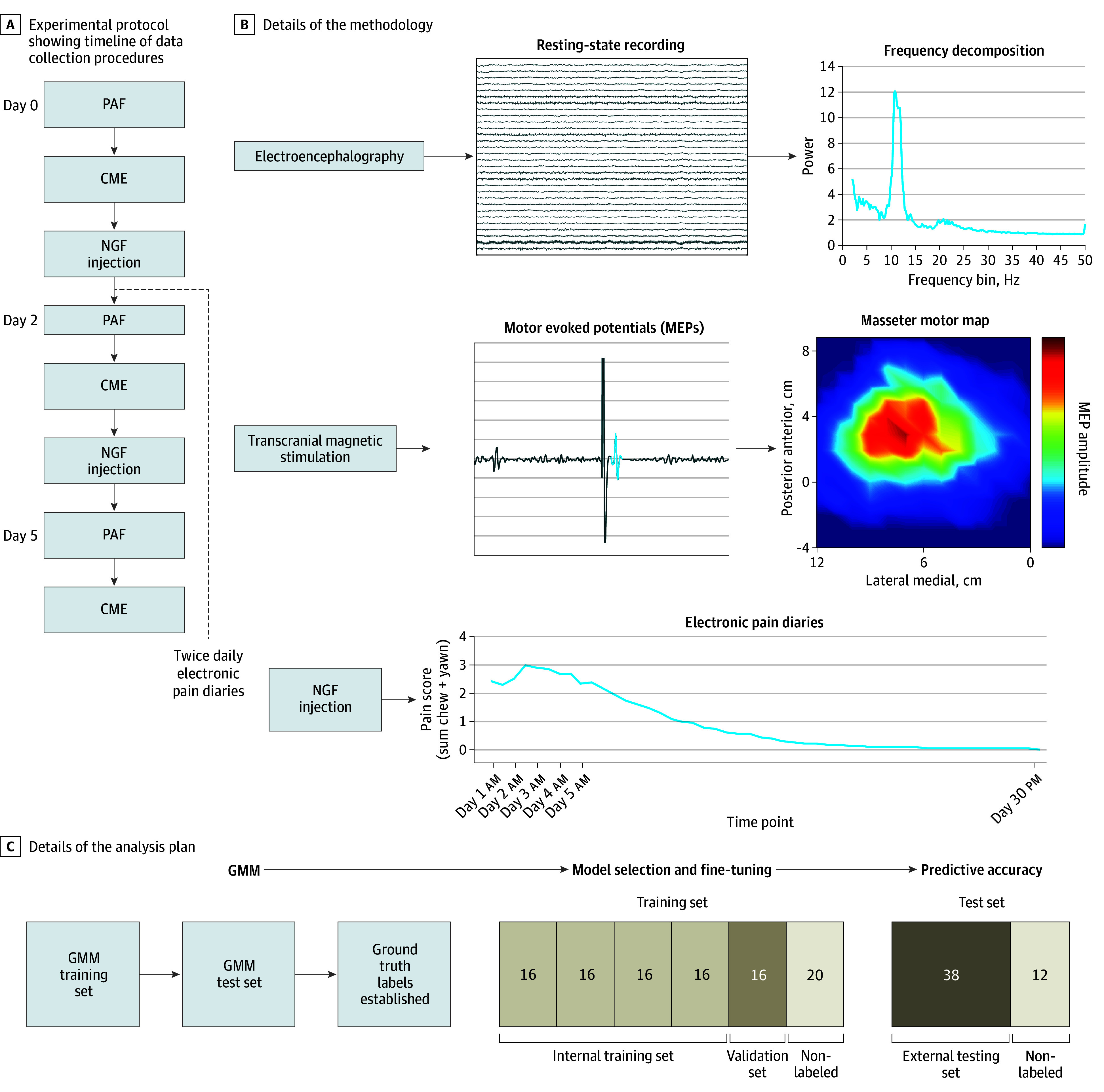
Study Methodology, Including Study Design, Data Collection Procedures, and Analysis Plan A, We measured peak alpha frequency (PAF) and corticomotor excitability (CME) at each session. At the end of 2 sessions, an injection of nerve growth factor (NGF) was administered to the right masseter muscle. B, Refer to the “Methods” section for details of how PAF was computed from the electroencephalography recording and how CME was computed from the transcranial magnetic stimulation mapping. From days 1 through 30, electronic diaries measuring jaw pain were sent to participants at 10 am and 7 pm each day. The pain score was quantified as the sum of scores for pain on chewing and yawning. C, Refer to the “Methods” section for details about the nested control-test scheme, growth mixture modeling (GMM), and machine learning models.

Outcomes were collected over a period of 30 days. Participants attended the laboratory on day 0, 2, and 5. Baseline questionnaire data were collected on day 0. Pressure pain thresholds (eFigure 4 in Supplement 1), PAF, and CME were measured on day 0, 2, and 5. PAF was obtained via a 5-minute eyes-closed resting-state electroencephalography recording from 63 electrodes. Sensorimotor PAF was computed by identifying the component in the signal (transformed by independent component analysis) that had a clear alpha peak (8-12 Hz) on frequency decomposition and a scalp topography suggestive of a sensorimotor source. CME was obtained using transcranial magnetic stimulation (TMS) mapping; single pulses of TMS delivered to the left M1, and motor evoked potentials (MEPs) recorded from the right masseter muscle using electromyography electrodes. TMS was delivered at each site on a 1 cm–spaced grid superimposed over the scalp, and a map of the corticomotor representation of the masseter muscle was generated (eFigure 6 in Supplement 1). Corticomotor excitability was indexed as map volume, which is calculated by summing MEP amplitudes from all “active sites” on the grid. NGF was injected into the right masseter muscle at the end of the day 0 and 2 laboratory sessions. Electronic pain diaries were collected from days 1 to 30 at 10 am and 7 pm each day, where participant rated their pain (0-10) during various activities. Pain on functional jaw movement is a key criterion for the diagnosis of temporomandibular disorders,^30^ and pain during chewing and yawning are higher compared with other activities after an NGF injection to the masseter muscle.^28,31^ As such, the primary outcomes were pain on chewing and yawning. The protocol and methodology are shown in Figure 1A and B.

### Statistical Analysis

#### Division of the Data

Analysis was conducted in R version 4.2.2, MATLAB version 2022b, and Python version 3.1.2, with code publicly available.^32^ Figure 1C details the analysis plan. We adopted a nested control-test scheme by partitioning 150 participants into the first 100 (training set) and second 50 (test set) individuals to participate in the study.

#### Growth Mixture Modeling

We used growth mixture modeling (GMM) in R^33,34,35^ to form 2 classes: high and low pain sensitive. For this categorization, we used the sum of data for pain on chewing and yawning and pain data from days 1 through 7, as this was the time frame when pain was most prominent (eFigure 3 in Supplement 1). As such, participants would more reliably fall into high and low pain-sensitive classes during this time frame. The first and last 40 participants (80 in total) in the training set, based on the ordering of probabilities of the pain intensity trajectory belonging to one of the classes, were labeled as high and low pain sensitive. The trained GMM model, once established, was locked and used to label the test set. Consequently 38 of 50 test set participants were labeled, with a different proportion of high and low pain sensitive (24 high and 14 low pain) compared with the training set since the classifications were based on the probability thresholds established in the training set. These labels were recorded for subsequent comparison with the predicted labels produced by the trained machine learning model.

#### Machine Learning Model Selection and Fine-Tuning

We used 5 machine learning models on the labeled training set: logistic regression, random forest, gradient boosting, support vector machine, and neural network. The dependent variable was pain sensitivity label (high/low) identified from the GMM, and independent variables were sensorimotor PAF and change in CME (ΔCME): the latter was typified as facilitator and depressor, depending on whether they showed an increase or decrease in map volume on day 5 relative to day 0, respectively. For each model, we identified optimized parameters through 5-fold cross-validation: we randomly divided the 80 participants into an internal training set of 64 participants (consisting of 4 equal folds of 16) and a validation set of 16. The optimized models in the internal training set were then used to predict labels in the validation set to facilitate model selection. The model with the best performance (AUC) on the validation set was then locked in.

#### Test Set Prediction

The locked machine learning model was assessed on the test set. The participant identifications in this set did not coincide with those in the pain diary data, thereby preserving the double-blind nature of the analysis. By using the ground-truth labels (shuffled), predicted labels (unshuffled), and the shuffling order for the test set, we were able to evaluate the model’s performance by comparing the reordered predicted labels against the ground-truth labels established by the GMM. Performance was assessed via ROC area under the curve (AUC), with 95% CIs reported. AUC values from 0.7 to 0.8, 0.8 to 0.9, and 0.9 to 1.0 were considered acceptable, excellent, and outstanding, respectively.^29^

## Results

The PREDICT trial enrolled 159 healthy participants, with 150 participants remaining after dropouts. There were 70 female participants and 89 males; the mean (SD) age was 25.1 (6.1) years.

### Good to Excellent Test-Retest Reliability

PAF and ΔCME showed good to excellent test-retest reliability across sessions (eFigures 5 and 7 in Supplement 1).

### Outstanding Performance on the Training Validation Set

**Figure 2.**
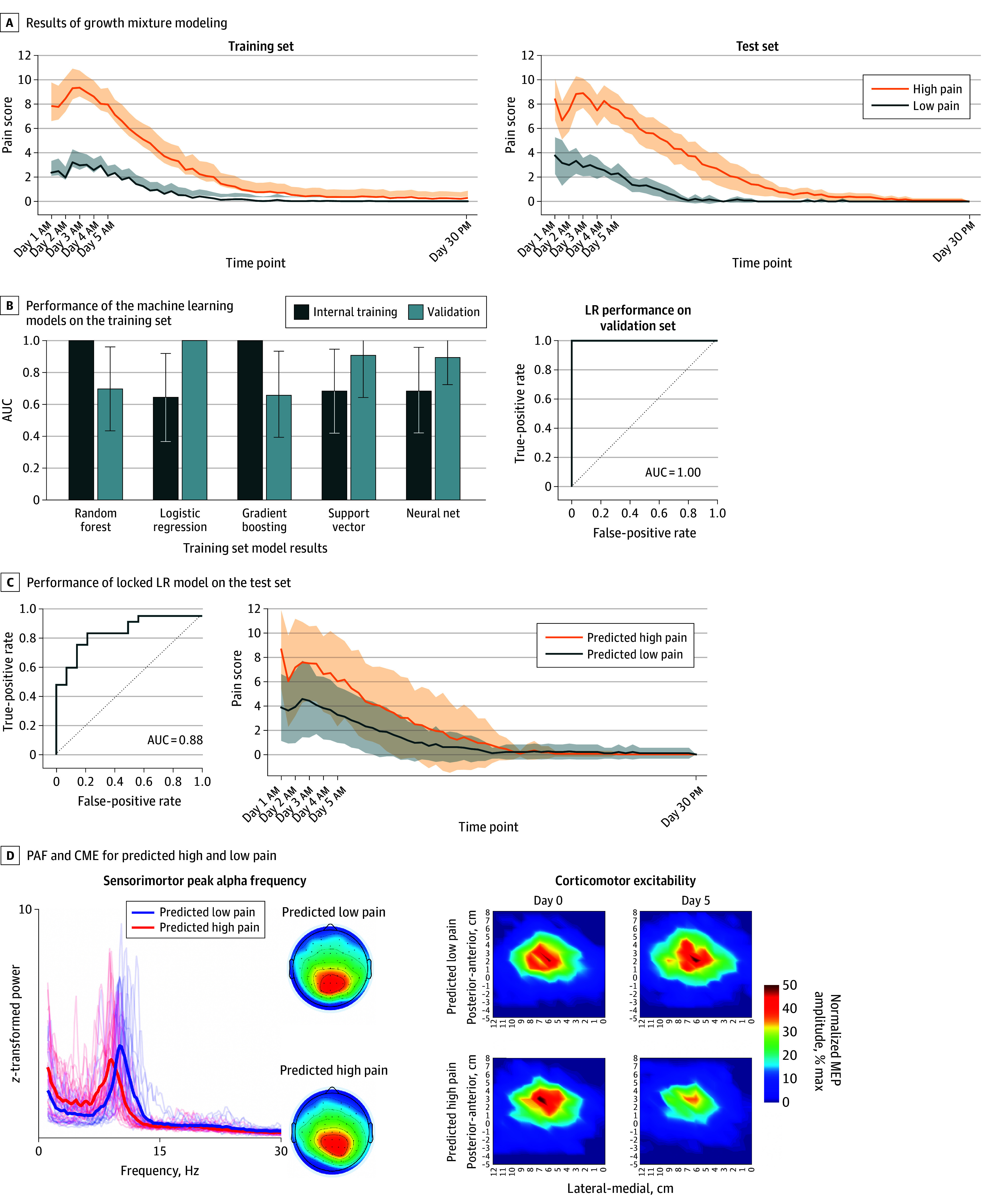
Performance of the Combined Peak Alpha Frequency (PAF) and Corticomotor Excitability (CME) Biomarker on the Training and Test Sets A, The growth mixture modeling categorized 80 participants in the training set and 38 participants in the test set as high or low pain sensitive. B, Performance of different machine learning models for the internal training and validation sets. C, Performance of the locked logistic regression (LR) model when applied to the test set. D, Characteristics of sensorimotor PAF and CME for individuals predicted to have high or low pain in the test set. The left panel shows the individual and mean *z*-transformed spectral plots and topography of the sensorimotor alpha component on day 0. The right panel displays the mean motor cortex maps on day 0 and day 5 showing normalized motor evoked potential (MEP) amplitude (expressed as a percentage of the maximal MEP amplitude). Error bars and shading indicate 95% CIs; AUC, area under the curve.

Figure 2A shows the pain scores for participants in the training and test set classified as high and low pain sensitive. Figure 2B shows the performances of the models across the internal training and validation sets. Logistic regression was the winning classifier based on its outstanding performance (AUC = 1.00; 95% CI, 1.00-1.00) when applied to the validation set, with slower PAF and CME depression predicting higher pain with regression coefficients of −1.25 and −1.27 for PAF and CME, respectively.

### Excellent Performance on the Test Set

When the locked logistic regression model was applied to the test set, performance (Figure 2C, left) was excellent (AUC = 0.88; 95% CI, 0.78-0.99). The optimal probability threshold for being classified as high pain sensitive was 0.40, with an associated sensitivity of 0.875 and specificity of 0.79. Applying this 0.40 probability threshold to our data, to be labeled as high pain sensitive, a facilitator would need a PAF less than 9.56, and a depressor would need a PAF less than 10.57. Figure 2C (right) shows the differences in pain scores between participants predicted to have high or low pain. Visually slower PAF can be observed in individuals predicted to have high vs low pain sensitivity (Figure 2D). This was statistically significant according to a 2-sample *t* test (*t*_48_ = 5.8, *P* < .001). Moreover, a decrease in CME can be observed within the masseter motor maps in individuals predicted to have high pain (Figure 2E), whereas those predicted to have low pain exhibited an increase in CME. The differences in ΔCME between these groups was statistically significant (*t*_48_ = 2.81, *P* = .007).

### Benefit of a Combined Signature

We reran the models to determine whether the combined PAF/CME signature outperformed each measure individually (eFigure 10 in Supplement 1). The performance of the PAF-only logistic regression model on the training validation and test set were respectively outstanding (AUC = 0.95; 95% CI, 0.84-1.00) and excellent (AUC = 0.83; 95% CI, 0.70-0.96). The performance of the CME-only logistic regression model for the training validation and test set were respectively excellent (AUC = 0.88; 95% CI, 0.69-1.00) and acceptable (AUC = 0.75; 95% CI, 0.60-0.91).

### Model Performance Not Improved by Covariate Inclusion

**Figure 3.**
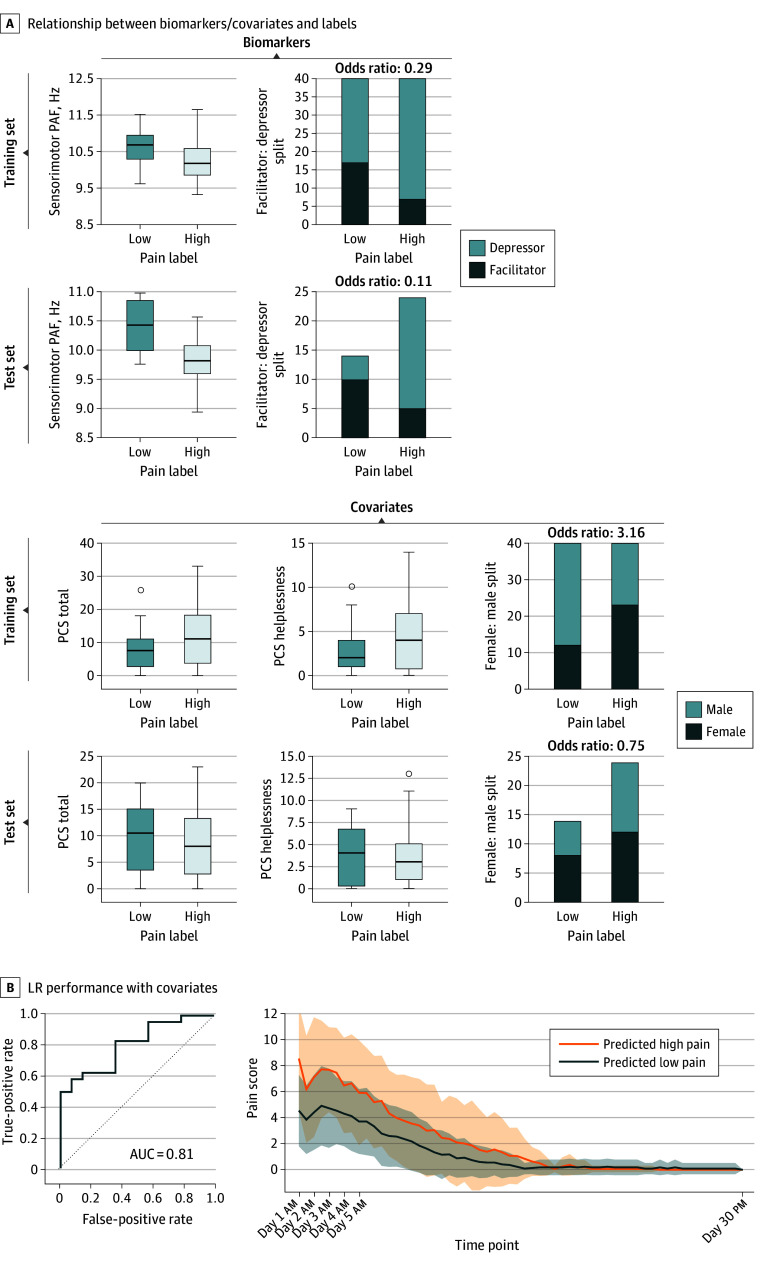
Performance of the Combined Peak Alpha Frequency (PAF) and Corticomotor Excitability (CME) Biomarker on Training and Test Sets When Including Covariates A, Summary of biomarker and covariates for participants labeled as high or low pain sensitive. Pain labels were identified from growth mixture modeling. A lower odds ratio means a lower probability of individuals with high pain sensitivity belonging to the facilitator or female categories. B, Performance of the logistic regression (LR) model applied to the test set when including both biomarkers and covariates. Pain score was the sum of scores for pain on chewing and yawning; shading indicates 95% CIs. AUC indicates area under the curve; PCS, Pain Catastrophizing Scale.

We evaluated the performance of the biomarker combined with covariates. As there were many variables, we applied feature selection, that is, filtering features by inspecting *P* values when associating predictors and labels, and using parameter tuning to optimize the coefficients associated with the filtered features. Five features were subsequently selected and optimized: sensorimotor PAF, CME, sex, Pain Catastrophizing Scale (PCS) total score, and PCS helplessness score. The associations between labels and biomarkers/covariates in the training vs test set and performance of the models are shown in Figure 3A and B. When including these 5 features, the performance of the logistic regression model (regression coefficients of −0.86, −0.69, 0.64, 0.02, and 0.06 for PAF, CME, sex, PCS total score, and PCS helplessness score, respectively) was outstanding (AUC = 1.00; 95% CI, 1.00-1.00) and excellent (AUC = 0.81; 95% CI, 0.67-0.95) for the training validation and test set, respectively. Thus, the model with biomarkers only outperformed the model including covariates.

### Results Reproducible Across Methodological Choices

**Figure 4.**
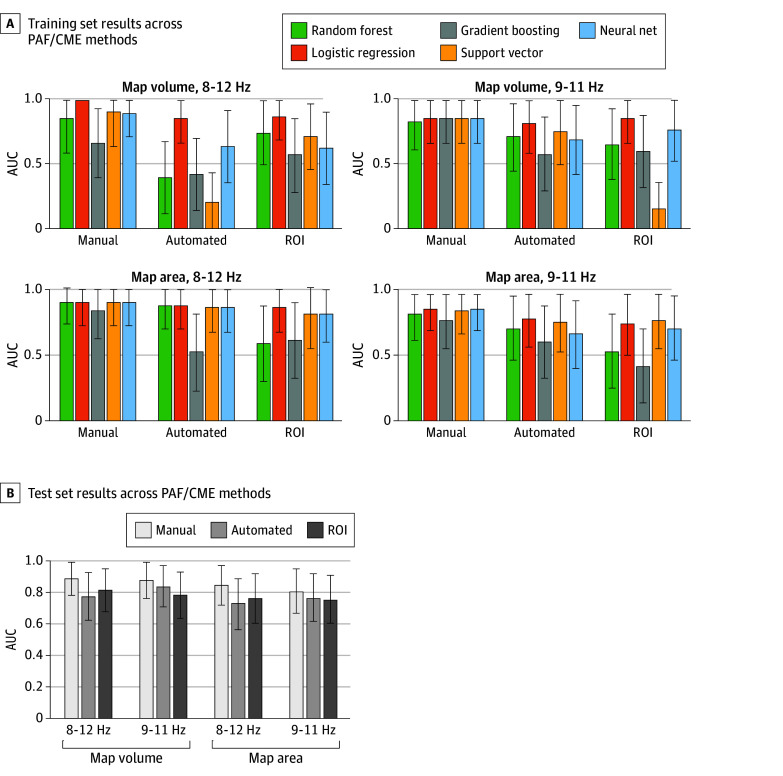
Performance of the Combined Peak Alpha Frequency (PAF) and Corticomotor Excitability (CME) Biomarker on Training and Test Sets Across PAF/CME Calculation Methods A, Performance of each machine learning model on the training validation set across different PAF/CME calculation methods. B, Performance of the locked logistic regression (LR) model applied to the test set across different PAF/CME calculation methods. AUC indicates area under the curve; ROI, region of interest.

To determine whether our results were robust across different methodological choices, we repeated the analysis using different PAF calculation methods, including component-level data (with the sensorimotor component chosen manually or using an automated script) vs sensor-level data (with a sensorimotor region of interest), using different frequency windows (8-12 Hz vs 9-11 Hz) and using different CME calculation methods (map volume vs map area). We found that, regardless of the choices, logistic regression was the best or equal-best–performing model when applied to the training validation set (Figure 4), with AUCs varying from acceptable (AUC = 0.77) to outstanding (AUC = 1.00). When the locked models were applied to the test set, performance varied from acceptable (AUC = 0.73) to excellent (AUC = 0.88) (Figure 4). Lastly, excellent performance was demonstrated when the data were analyzed 3 other ways (eFigures 11-13 in Supplement 1): where GMM pain labels were established using the whole 30 days rather than the first 7 days (training validation AUC = 0.84; 95% CI, 0.64-1.00; test AUC = 0.89; 95% CI, 0.79-0.99), and when missing pain diary data was not imputed (training validation AUC = 0.81; 95% CI, 0.6-1.00; test AUC = 0.89; 95% CI, 0.79-0.99).

## Discussion

A full-scale analytical validation of the PAF and CME biomarker signature was conducted using a prolonged pain model. In an initial training set (n = 100), we found that a logistic regression was the optimal classifier based on its outstanding performance (AUC = 100%), with slower PAF and CME depression predicting higher pain. When this model was applied to an independent test set, the AUC was excellent (AUC = 88%). PAF/CME showed good to excellent test-retest reliability, and results were reproduced across a range of methodological parameters. Inclusion of covariates did not improve model performance, suggesting the model including biomarkers only was more robust. Overall, the combination of sample size, pain model validity, and biomarker accuracy, reproducibility, and reliability suggests the PAF/CME biomarker signature has substantial potential for clinical translation.

Our results suggest that individuals who have slow PAF before an anticipated prolonged pain episode and show corticomotor depression during a prolonged pain episode are more likely to experience higher pain (eFigures 8 and 9 in Supplement 1). Model performance was higher combining the two, suggesting consideration of both ascending sensory and descending motor pain processing mechanisms provides more information regarding pain sensitivity. Given that higher acute pain predicts the development of chronic pain,^24^ our results suggest that PAF and CME could potentially be used as susceptibility biomarkers for the transition from acute to chronic pain.

Several aspects of our study stand out. The first is sample size: with recent advancements in machine learning, it has become possible to conduct analytical validation of pain biomarkers. However, deep learning requires a large amount of labeled samples to conduct rigorous training on validation and test sets.^8^ Unfortunately, many pain susceptibility biomarker studies have not been sufficiently sampled to adopt such approaches,^9,10^ and the ones that have used machine learning failed to reach the sample sizes similar to that of the present study.^1,2^

Another strength of our findings is reproducibility. Previous work has shown similar associations between slower PAF and higher upper limb pain, postoperative thoracic pain, and chronic pain in various body regions,^17,19,20^ as well as CME depression and higher upper limb pain, chronic patellofemoral pain, and development of chronic low back pain.^22,23,37,38^ The present study replicated these results in a model of jaw pain, suggesting the biomarker signature may be generalizable to pain more broadly. Note that some studies have not shown a negative relationship between PAF and pain sensitivity^39,40^ or a positive relationship between CME depression and pain sensitivity.^31^ However, these studies were not sufficiently sampled to conduct analytical validation of the kind presented in this study. Nonetheless, the mixed findings could also arise from differences in methodological choices in the estimation of PAF, for example, frequency windows^40^ and use of sensor vs component space data.^41^ and estimation of CME, for example, map volume^22^ vs area.^31^ For this reason, we repeated the main analysis using different methodological choices and found at least acceptable AUCs, supporting the reproducibility of our results.

The PAF/CME measures demonstrated good to excellent reliability. Reliability is a desirable characteristic that assists in the widespread application of pain biomarkers.^8^ We found that participants exhibit stable PAF across days despite the presence of pain and even when considering different methodological factors that may influence the reliability such as preprocessing pipeline, recording length, and frequency window.^14^ Indeed, reliable PAF was found with a recording length as short as 2 minutes and minimal data preprocessing. We also showed that those who show CME depression on day 2 are also likely to show CME depression on day 5 (and vice versa for those who show CME facilitation). This was shown even when an automated method of determining MEP amplitude on each trial was applied. Thus, our work shows not only that PAF and CME can predict pain, but also the relative ease with which reliable PAF/CME data can be obtained is promising for subsequent clinical translation.

Another strength of this study is our pain model. While other pain biomarker studies have shown promising results, these studies were restricted to pain models using transient painful stimuli lasting seconds to minutes.^11,12,13^ The brief nature of the painful stimuli questions the external validity of these findings and limits generalizability to clinical populations. In contrast, the present study used a prolonged pain model lasting weeks. Several other studies have shown that injections of NGF to the neck, elbow, or masseter muscles can mimic symptoms of clinical neck pain,^42^ chronic lateral epicondylalgia,^27^ and temporomandibular disorders,^28^ respectively. Thus, the observed relationships between PAF/CME and pain in the present study show promise in terms of clinical applicability.

Lastly, the PAF/CME biomarker demonstrated high performance. A previous study found that connectivity between medial prefrontal cortex and nucleus accumbens in 39 patients with subacute low back pain (pain duration, 6-12 weeks) could predict future pain persistence at approximately 7, 29, and 54 weeks, with AUCs of 67% to 83%.^1^ Another study on 24 patients with subacute low back pain showed that white matter fractional anisotropy measures in the superior longitudinal fasciculus and internal capsule predicted pain persistence over the next year, with an AUC of 81%.^2^ Although the present study did not directly assess the transition to chronic pain, our AUCs of 100% (validation set) and 88% (test set) appear comparatively high. We therefore encourage future clinical studies to determine whether PAF/CME can predict the transition from acute to chronic pain.

### Limitations

There were some limitations to our study. Our study used a cohort of healthy participants with strict inclusion/exclusion criteria and an experimental pain model. While this may limit generalizability to clinical populations, the use of a standardized sample/design is a requirement of preclinical analytical validation and an essential first step in the discovery pipeline toward a clinical biomarker signature. There is already evidence that the proposed biomarker is generalizable to clinical contexts. A recent study showed that individuals with slower PAF experienced more pain after a thoracotomy.^20^ Furthermore, individuals with lower CME during the acute stages of low back pain were more likely to develop chronic pain at the 6-month follow-up.^36^ This suggests PAF and CME shows promise in being used in preoperative, postoperative, and postinjury contexts to classify individuals with high or low pain sensitivity.

## Conclusions

This study found that a novel biomarker signature comprised of PAF and CME distinguishes individuals with high or low pain sensitivity during prolonged jaw pain with an excellent AUC of 88% in an independent test set. The combination of biomarker accuracy, reproducibility, reliability, and pain model validity suggests high potential for clinical translation, particularly in predicting the transition from acute to chronic pain.

## Notes

Supplement 1.
eMethods
**eFigure 1.** Alpha band power of each ICA component was compared against a sensorimotor template generated from previous work **eFigure 2.** Pipeline for automated selection of sensorimotor alpha component
eResults
**eTable 1.** Baseline questionnaire results **eTable 2.** Race demographics **eFigure 3.** Means for Pain Diaries **eFigure 4.** Means for Pressure Pain Thresholds **eFigure 5.** PAF Test-Retest Reliability **eFigure 6.** TMS Motor Maps **eFigure 7.** CME Test-Retest Reliability **eFigure 8.** Pain Diary Separated by PAF/CME Classifications **eFigure 9.** Comparison of pain scores for fast vs. slow PAF and facilitator vs. depressor **eFigure 10.** Model Performance of PAF/CME markers alone **eFigure 11.** Model Performance without linear interpolation of pain data **eFigure 12.** Model Performance when using the Days 1-30 for pain labelling **eFigure 13.** Model performance when including CME as a continuous variable
eReferences

Supplement 2. Data sharing statement
