# Supplementary material for: Predicting Individual Pain Sensitivity Using a Novel Cortical Biomarker Signature
Source: JAMA Neurol. 2025 Jan 27;82(3):237–46. doi: 10.1001/jamaneurol.2024.4857 (PMC11773403; doi:10.1001/jamaneurol.2024.4857)
Supplement: Supplement 2. — Data sharing statement [file jamaneurol-e244857-s002.pdf]

Data Sharing Statement

Chowdhury. Novel Cortical Biomarker Signature as a Predictor of Individual Pain Sensitivity. *JAMA Neurol*. Published January 27, 2025. doi:10.1001/jamaneurol.2024.4857

Data
Data available: Yes
Data types: Deidentified participant data
How to access data: TMS_Data: https://osf.io/r3m9g/ EEG_Data: https://openneuro.org/datasets/ds005486/versions/1.0.0
When available: With publication

Supporting Documents
Document types: Statistical/analytic code
How to access documents: https://github.com/DrNahianC/PREDICT_Scripts
When available: With publication

Additional Information
Who can access the data: Publicly available
Types of analyses: Code supporting all analysis in the paper
Mechanisms of data availability: Publicly available link
